# Supplementary material for: Identification of photoperiod-induced specific miRNAs in the adrenal glands of Sunite sheep (Ovis aries)
Source: Front Vet Sci. 2022 Jul 22;9:888207. doi: 10.3389/fvets.2022.888207 (PMC9354845; doi:10.3389/fvets.2022.888207)
Supplement: Supplementary file 1 [file Data_Sheet_1.ZIP › Supplementary file/miRDeep2 file.zip!/miRDeep2 file/Novel_Structure/SP42A3/Novel_598.pdf]

Provisional ID	:	NC_019461.2_10222
Score total	:	1.5
Score for star read(s)	:	-1.3
Score for read counts	:	-1.3
Score for mfe	:	2.6
Score for randfold	:	1.6
Score for cons. seed	:	
Total read count	:	9
Mature read count	:	4
Loop read count	:	2
Star read count	:	3

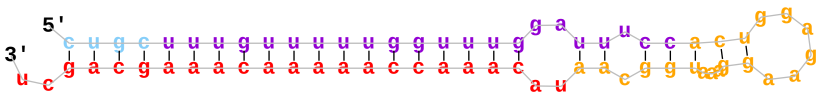

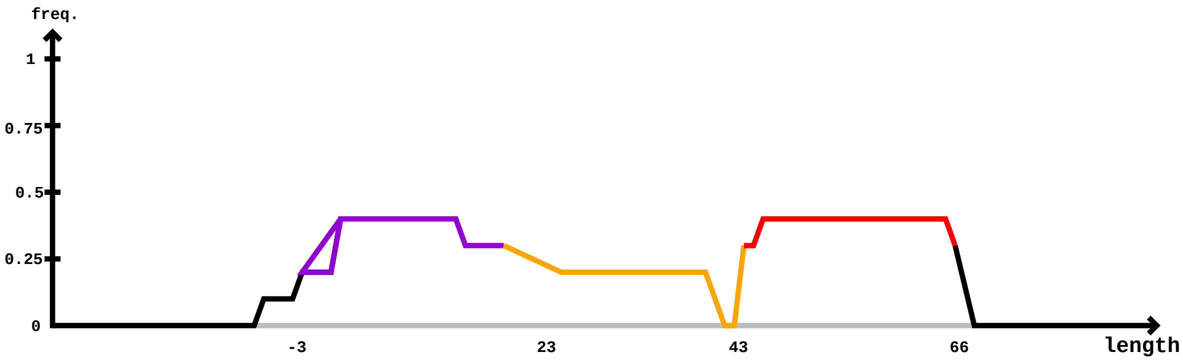


```
                                  Star                                      Mature
5'- gaucuaaacgggcgggguuauuagcugcuuuguuuuugguuuggauuuccacuggagaaggaaauggcaauacaaaccaaaaacaaagcagcuaaaauaaccccgccuguuua   -3'   obs
    gaucuaaacgggcgggguuauuagcugcuuuguuuuugguuuggauuuccacuggagaaggaaauggcaauacaaaccaaaaacaaagcagcuaaaauaaccccgccuguuua         exp
    ....(((((((((((((((((((((((((((((((((((((((..((.(((((......))...))).))..))))))))))))))))))))))..)))))))))))))))))    reads   mm   sample
    ....................uuagcugcuuuguuuuugguu........................................................................    1       0    seq
    ........................cugcuuuguuuuugguuuggauu..................................................................    1       0    seq
    ............................uuuguuuuugguuuggauuucc...............................................................    2       0    seq
    .................................................cacuggagaaggaaaugg..............................................    1       0    seq
    .................................................cacuggagaaggaaauggA.............................................    1       1    seq
    ......................................................................uacaaaccaaaaacaaagcagc.....................    1       0    seq
    ......................................................................uacaaaccaaaaacaaagcagcu....................    2       0    seq
    ........................................................................caaaccaaaaacaaagcagcu....................    1       0    seq
```
